# Clinical implications of reverse total shoulder arthroplasty with an os acromiale: a systematic review

**DOI:** 10.1016/j.xrrt.2025.01.002

**Published:** 2025-01-30

**Authors:** Lawrence Wengle, Andrew Kucey, Usama Saleh, Amr Elmaraghy

**Affiliations:** a Department of Orthopedic Surgery, St. Joseph’s Health Centre, University of Toronto, Toronto, ON, Canada; b Division of Orthopaedic Surgery, Department of Surgery, University of Toronto, Toronto, ON, Canada; c Department of Orthopaedic Surgery, Memorial University of Newfoundland, St. John’s, NL, Canada; d Medcare Orthopaedics & Spine Hospital, Dubai, United Arab Emirates

**Keywords:** Os acromiale, Acromion, Mesoacromion, Acromial tilt, Reverse shoulder arthroplasty, Total shoulder arthroplasty

## Abstract

**Background:**

Os acromiale is defined as a developmental failure of fusion of one of the primary ossification centers of the acromion. This anatomic variant can be identified in the presentation and workup of patients with various shoulder pathologies. Reverse total shoulder arthroplasty (rTSA) is a common surgical procedure for a multitude of underlying conditions. The purpose of this study was to conduct a systematic review of the literature to determine the clinical implications of rTSA in those with os acromiale.

**Methods:**

This systematic review was conducted according to the Preferred Reporting Items for Systematic Reviews and Meta-Analyses: the PRISMA checklist. In April 2024, the following online databases were accessed: PubMed, Embase, and Cochrane. All clinical studies assessing os acromiale in rTSA were considered for inclusion and evaluated.

**Results:**

The initial search result provided 569 studies to be assessed. After careful screening, 4 studies were included in this systematic review. A total of 573 patients undergoing rTSA with underlying os acromiale were included in this review. The prevalence of os acromiale in patients undergoing rTSA ranged from 5% to 22%. All patients had improvements in patient reported outcome measures with minimal complications. The most common radiographic finding was inferior tilting of the os acromiale.

**Conclusion:**

The presence of os acromiale does not appear to have a negative impact on the clinical outcomes after surgery and rTSA remains a safe and effective treatment option.

Os acromiale is defined as a developmental failure of fusion of one of the three primary ossification centers of the acromion.^10^ This can be further characterized into the subtypes of preacromion, mesoacromion, or meta-acromion depending on which center is involved, with mesoacromion being the most common (Fig. 1).^3^ The incidence of os acromiale has been reported to range from 1% to 15% in the literature.^17^ While most cases of os acromiale are asymptomatic and discovered as an incidental radiographic finding, some studies have suggested it may be a cause of pain and a mechanical contributor towards various shoulder pathologies.^6^^,^^12^^,^^15^ For example, subacromial impingement and end-stage rotator cuff disease are suggested to be more common in those with os acromiale.^12^ Thus, it is likely that some patients presenting for surgical treatment of their various shoulder pathologies will have the presence of this acromial morphology.

**Figure 1:**
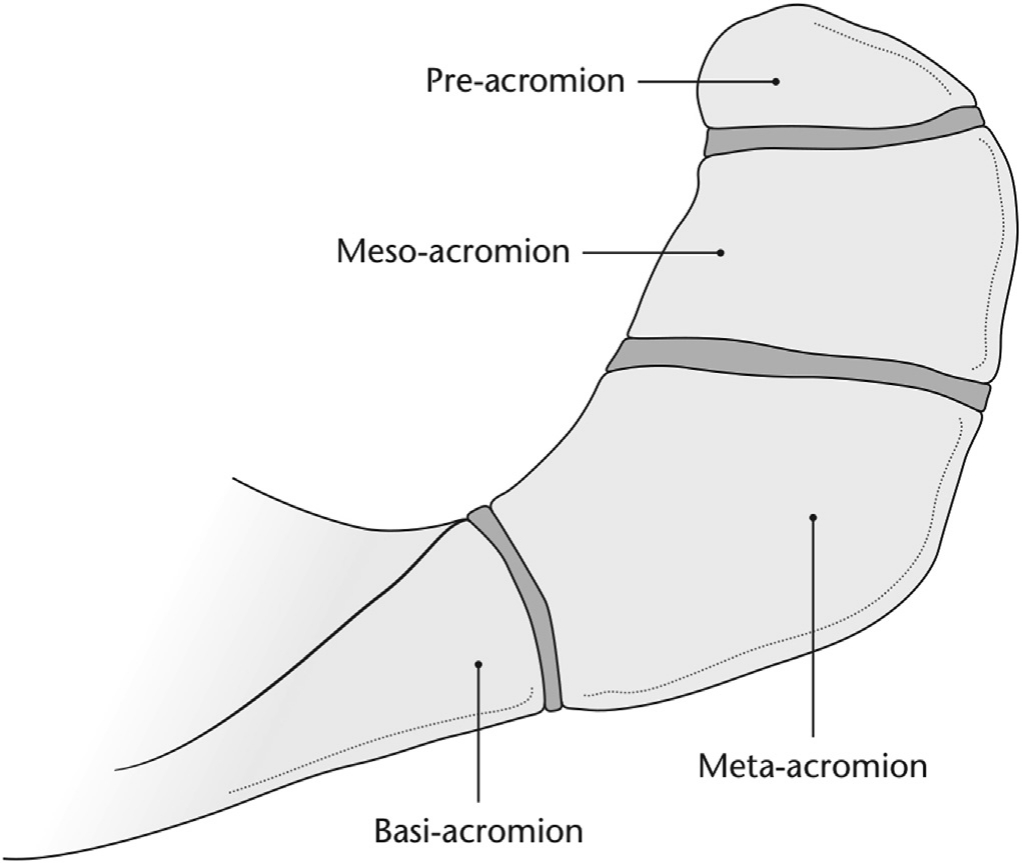
Acromial physes and the resultant anatomical subtypes of os acromiale. Illustration provided by Antbits Ltd.^8^

Reverse total shoulder arthroplasty (rTSA) is a common surgical procedure for a multitude of underlying conditions. It involves the replacement of the humeral head and native glenoid with prosthetic implants that provide a more distal and medial center of rotation compared to the native glenohumeral joint, thereby improving the mechanical advantage of the deltoid lever arm.^13^ The construct also places the proximal humerus more distal and lateral, increasing the active deltoid tension for improved shoulder function.^9^ There are a number of factors that should be considered for those undergoing rTSA in the presence of an os acromiale. It has been proposed that increased deltoid tension on an os acromiale can lead to further displacement or a stress fracture resulting in pain.^2^ Furthermore, the presence of an os acromiale may alter the normal biomechanics of an rTSA leading to worse clinical outcomes. However, other studies have claimed that the preoperative diagnosis of os acromiale has no negative impact on the clinical outcomes of this patient population.^2^

The purpose of this study was to conduct a systematic review of the literature to determine the clinical implications of rTSA in those with os acromiale. Specifically, our goal was to (1) evaluate outcome measures in the literature and (2) characterize any surgical considerations in this population. We hypothesize that rTSA is a safe and effective treatment option for patients with shoulder pathology, despite the presence of an os acromiale.

## Materials and methods

### Search strategy

This systematic review was conducted according to the Preferred Reporting Items for Systematic Reviews and Meta-Analyses: the PRISMA checklist.^11^ The endpoints of the initial search followed the PICO framework:

• P (population): patients with os acromiale;
• I (intervention): rTSA;
• C (comparison): patients with normal acromial ossification;
• O (outcomes): clinical scores, radiographic findings, surgical considerations.

### Literature search

Two independent authors (L. W., A. K.) performed the literature search. In April 2024, these online databases were accessed: PubMed, Embase, and Cochrane. The following keywords were used in combination: os acromiale, acromion, reverse shoulder arthroplasty, and total shoulder arthroplasty. An initial title and abstract review was performed prior to accessing the full-text of the articles of interest. The bibliographies of the included studies were also screened. Revman software (The Cochrane Collaboration, London, United Kingdom) was utilized to collate and screen articles. Disagreements between the authors were mutually debated and solved collectively as a group.

### Eligibility criteria

All clinical studies assessing os acromiale in rTSA were considered for inclusion. According to the Oxford Centre of Evidence-Based Medicine,^4^ articles level of evidence I to IV were included in the present work. Articles published since 1980 were included. Reviews, case reports, single-arm studies, expert opinions, letters, editorials, and articles unavailable in English were excluded. Animal, in vitro, and cadaveric studies were also excluded. Only studies reporting quantitative data on the outcomes of interest were considered eligible.

### Outcomes of interest

Two independent authors (L. W., A. K.) performed the data extraction of the included studies. The following data were collected for each study: author and year of publication, type of study, population, and demographic details. The outcomes of interest were clinical scores, radiographic evaluations, and complications or adverse events related to the underlying pathology.

## Results

### Search result

**Figure 2:**
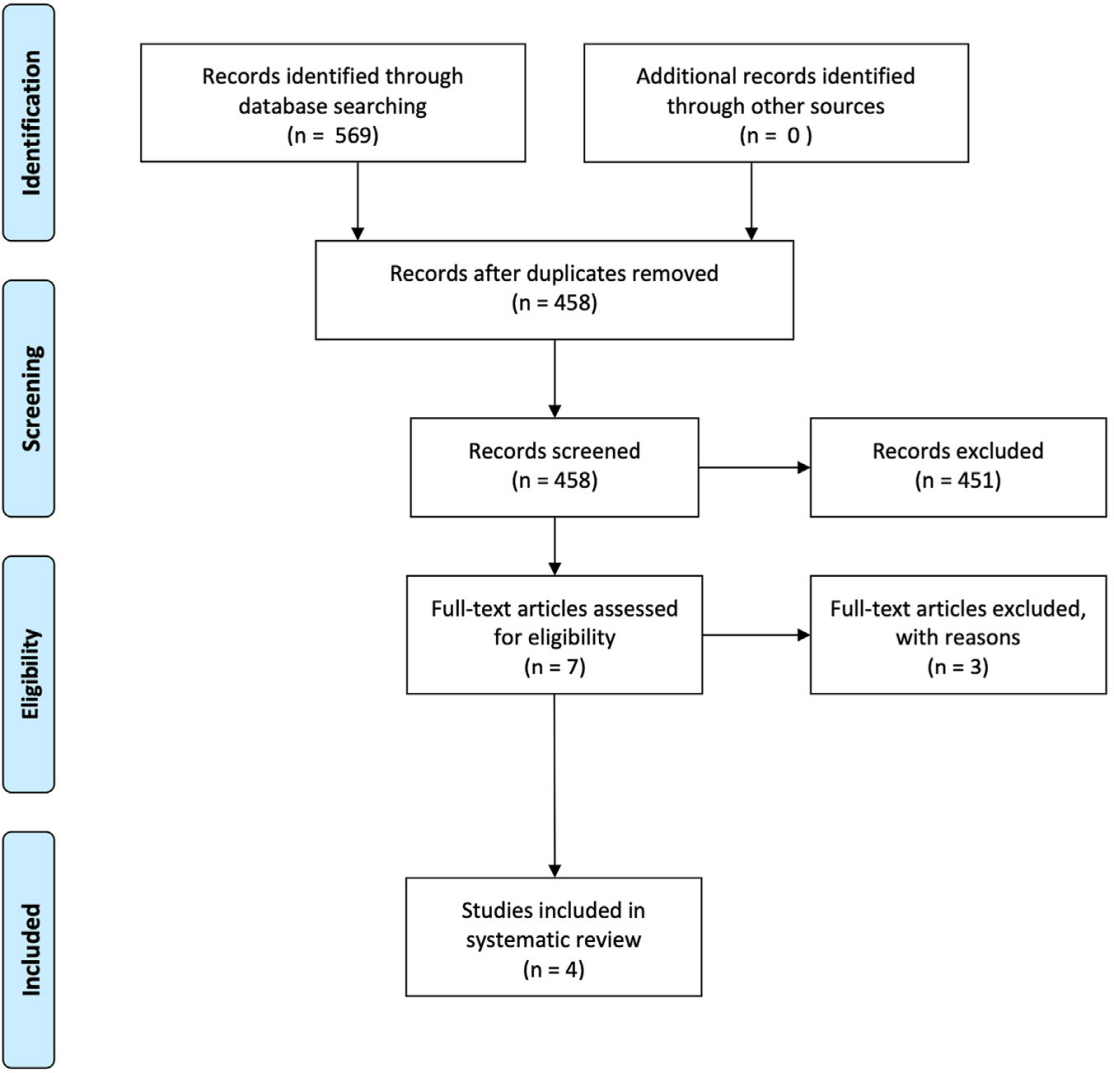
Preferred reporting items for systematic reviews and meta-analyses (PRISMA) flowchart of the literature search.

The initial literature search resulted in 569 studies from the three databases that were accessed. Of them, 111 duplicates were excluded. The studies were then screened by title and abstract to determine if they met the eligibility criteria. Seven papers were identified and proceeded to full text screening. Three of these did not meet our inclusion criteria, leaving 4 papers that were eligible for the systematic review.^2^^,^^5^^,^^7^^,^^16^ The flowchart of the literature search is shown in Fig. 2.

### Patient population

A total of 573 patients undergoing rTSA with underlying os acromiale were included in the four studies. The average age ranged from 69.6 to 74 year old, and 73% of the patients were female across the included studies. The most common indication for rTSA was rotator cuff arthropathy followed by irreparable rotator cuff tears.

### Prevalence of os acromiale

The prevalence of os acromiale in patients undergoing rTSA ranged from 5% to 22%. Ersen et al found that the prevalence of os acromiale was higher in patients who developed cuff tear arthropathy compared to the general population.^7^ The subtype of os acromiale was reported in all of the four studies with mesoacromion being the most common (81%), followed by preacromion (16%) and meta-acromion (3%).

### Functional outcomes

All of the studies showed improvements in patient-reported outcome measures after rTSA. Three of the studies demonstrated that patients with os acromiale undergoing rTSA had significant improvements in their postoperative Constant-Murley Score.^5^^,^^7^^,^^16^ Additionally, Aibinder et al found significant improvements in American Shoulder and Elbow Surgeons scores.^2^ Lastly, pain scores improved significantly after rTSA in all the studies included. There was no significant difference in patient-reported outcome measures between patients with and without os acromiale. When evaluating range of motion, all of the studies found that rTSA provided significant improvements in range of motion in patients with os acromiale compared to their preoperative baseline. However, Carpeggiani et al found that patients with os acromiale had significantly decreased active flexion compared with the controls of 104° ± 33° vs. 114° ± 33° (*P* = .03) at 1 year postoperatively and active abduction of 103° ± 37° vs. 121° ± 38° (*P* = .02) at 2 years postoperatively, respectively.^5^

Postoperative pain related to the os acromiale was reported in 4%-27% of patients. However, one study reported that this spontaneously resolved in the majority of cases after a mean of 33 months.^5^ Overall, there was no significant difference in postoperative complications between groups in all of the studies included.

### Radiographic findings

From a radiographic standpoint, the most common complication in all four studies was inferior tilting of the os acromiale which occurred in 28%-50% of patients. Scapular notching was noted in three of the studies and ranged from 17% to 40% of patients with underlying os acromiale. Implant loosening was reported by Carpeggiani to be 4.4% in this patient population.^5^ Lastly, the acromiohumeral distance was evaluated in one of the studies and found to be significantly shorter in patients with os acromiale.^7^ This was measured from the most lateral end of acromion to the top of the major tubercle. These findings are all summarized in Table I.

**Table I:** Methodology and results summary of the four studies included in the systematic review.

Study	Clinical focus	Study design (level of evidence)	Methods	Demographics	Primary outcome variables	Key findings	Primary conclusion
Walch et al 2009^15^	Acromial insufficiency in rTSA	Case-control (III)	Retrospective chart review and comparison of all rTSA performed at their institution between 1992 and 2003.	• 457 total rTSA performed • Preoperative acromial pathology identified in 45 patients ○ Os acromiale, N = 23 patients • Mean age: 72.3 • Male 22.5%, female 77.5% • Os acromiale subtype ○ Mesoacromion (100%)	• Active range of motion • PROMs ○ CMS score	• Preoperative acromial lesions did not negatively affect outcomes. • Os acromiale had a prevalence of 5% (23/457) • All os acromiale identified were mesoacromion • Patients with os acromiale had statistically higher Constant scores than those with normal acromial morphology	Preexisting acromial lesions including os acromiale, are not a contraindication for reverse shoulder arthroplasty.
Aibinder et al 2017^2^	rTSA in patients with os acromiale	Case Series (IV)	Retrospective chart review of all primary rTSAs performed at their institution between 2005 and 2013. Inclusion criteria: • Presence of os acromiale • Diagnosis of CTA • Minimum 2 year follow up after rTSA surgery	• 25 patients all with os acromiale in the setting of CTA • Mean age: 72 (46-84) • Male 68%, female 32% • Os acromiale subtype ○ Mesoacromion (80%) ○ Preacromion (12%) ○ Meta-acromion (8%)	• PROMs ○ Neer Pain Score ○ Modified Neer rating ○ ASES score • Active range of motion • Radiographic evaluation ○ Os acromiale tilting ○ Implant loosening ○ Scapular notching	• Improved pain and function in 24/25 patients after rTSA • Only 1 patient (4%) experienced pain related to os acromiale after rTSA • Os acromiale tilting noted in 28% of patients on postoperative imaging • No evidence of implant loosening or failure	The presence of os acromiale does not negatively impact the overall outcome of rTSA.
Ersen et al 2019^7^	rTSA in patients with os acromiale	Case-control (III)	Retrospective chart review and comparison of all rTSA performed at their institution between 2009 and 2015. Inclusion criteria: • Diagnosis of CTA • Minimum 2 year follow up after rTSA surgery Exclusion criteria: • rTSA for proximal humerus fracture • Revision rTSA	• 46 patients with RTSA for CTA ○ Os acromiale (N = 10 patients) ○ Without os acromiale (N = 36 patients) • Mean age: 70.8 (56-84) • Male 15%, female 85% • Os acromiale subtype ○ Mesoacromion (100%)	• PROMs ○ CMS Score ○ Q-DASH Score ○ VAS Score • Active range of motion • Radiographic evaluation ○ Acromiohumeral distance	• 22% of patients had os acromiale • Both groups showed significant functional outcome improvements after rTSA • Acromiohumeral distance was significantly shorter in patients with os acromiale • Os acromiale migrated distally in all patients with this condition	Os acromiale does not adversely affect the clinical outcomes of rTSA but the loose fragment can migrate distally postoperatively due to deltoid tension.
Carpeggiani et al 2020^5^	rTSA in patients with os acromiale	Case-control (III)	Retrospective comparison of patients that underwent rTSA at their institution between 2005 and 2016. Patients screened and matched based on the presence or absence of preoperative os acromiale. Case-controlled matching (max 1:3) criteria included: • Patient sex • Patient age • Indication for rTSA • Time of follow-up (minimum 1 year)	• Study group (presence of os acromiale) ○ N = 42 patients ○ Mean age (74 ± 9) • Control group (absence of os acromiale) ○ N = 132 patients ○ Mean age (72 ± 8) • Os acromiale subtype ○ Mesoacromion (67%) ○ Preacromion (31%) ○ Meta-acromion (2%) Sex distribution and indications for rTSA were matched and comparable between groups.	• PROMs ○ CMS score ○ SSV score • Active range of motion • Radiographic assessment ○ Type of os acromiale ○ Scapular notching ○ Postoperative displacement/tilting • Postoperative complications and revision surgeries	• Both groups showed significant improvements in PROMs and range of motion from preoperative levels to final follow up • No significant difference in PROMs between groups at final follow up • Patients with os acromiale had slightly decreased active flexion and abduction • Postoperative pain at the os acromiale occurred in 27% of cases and spontaneously resolved in the majority after a mean of 33 months • No significant difference in postoperative complications between groups	rTSA reliably restores patient satisfaction in individuals with os acromiale. Postoperative pain at the os acromiale is relatively common but typically resolves spontaneously over time.

*rTSA*, reverse total shoulder arthroplasty; *PROM*, Patient Reported Outcome Measure; *ASES*, American Shoulder and Elbow Surgeons; *CTA*, cuff tear arthropathy; *CMS*, Constant-Murley Shoulder Outcome Score; *Q-DASH*, QuickDASH score; *VAS*, visual analog scale; *SSV*, subjective shoulder value.

## Discussion

RTSA has become an increasingly popular and effective surgical treatment option for patients with various underlying shoulder conditions. However, the distalization of the shoulder’s native center of rotation and increase in deltoid tension raise theoretical concerns for patients with underlying os acromiale. Further displacement of the os, inferior tilting, pain, and loss of mechanical advantage have all been postulated as concerns in this condition.^2^ While multiple studies have investigated os acromiale in those undergoing rTSA, this is the first systematic review to evaluate this topic. It was determined that rTSA in the presence of os acromiale remains a safe and effective treatment option without any significant consequences on clinical outcomes.

Our study demonstrated a similar prevalence of os acromiale in patients undergoing rTSA compared to what has been quoted in the literature. However, Ersen et al found a slightly higher prevalence of 22% in those with underlying rotator cuff arthropathy.^7^ This may suggest that underlying os acromiale may predispose patients to developing rotator cuff arthropathy in the setting of cuff degeneration. It has been proposed that inferior tilting of the os fragment during contracture of the deltoid tendon can lead to subacromial impingement and higher wear patterns of the underlying rotator cuff.^1^ Additionally, females made up a large proportion of the patients included in this review. This may be an over representation of gender disparity given that most of the literature suggests no significant difference between males and females.^17^ Black ethnicity has been the most common correlation from a patient demographic standpoint but the studies included in this review did not include patient ancestry or racial data.^14^

The four studies included in this review consistently demonstrated improvements in patient reported outcome measures after rTSA. With a range of shoulder specific outcome scores including Constant-Murley Scores, American Shoulder and Elbow Surgeons, and Subjective Shoulder Value, patients with os acromiale were found to have significantly improved function after rTSA. Additionally, patient satisfaction was high in all studies. From an active range of motion standpoint, Carpeggiani et al found that patients with os acromiale had slightly decreased active flexion and abduction after surgery compared to control patients.^5^ Although this was found to be statistically significant, there was only a 10° change in active flexion and 18° change in active abduction identified. This difference may be due to the fact that the os acromiale can migrate distally after rTSA, which can lead to decreased deltoid muscle tension and mechanical advantage. While this is a consideration to discuss with os acromiale patients prior to surgery, the strong evidence from this review suggests that rTSA remains an effective treatment for patients with shoulder dysfunction.

**Figure 3:**
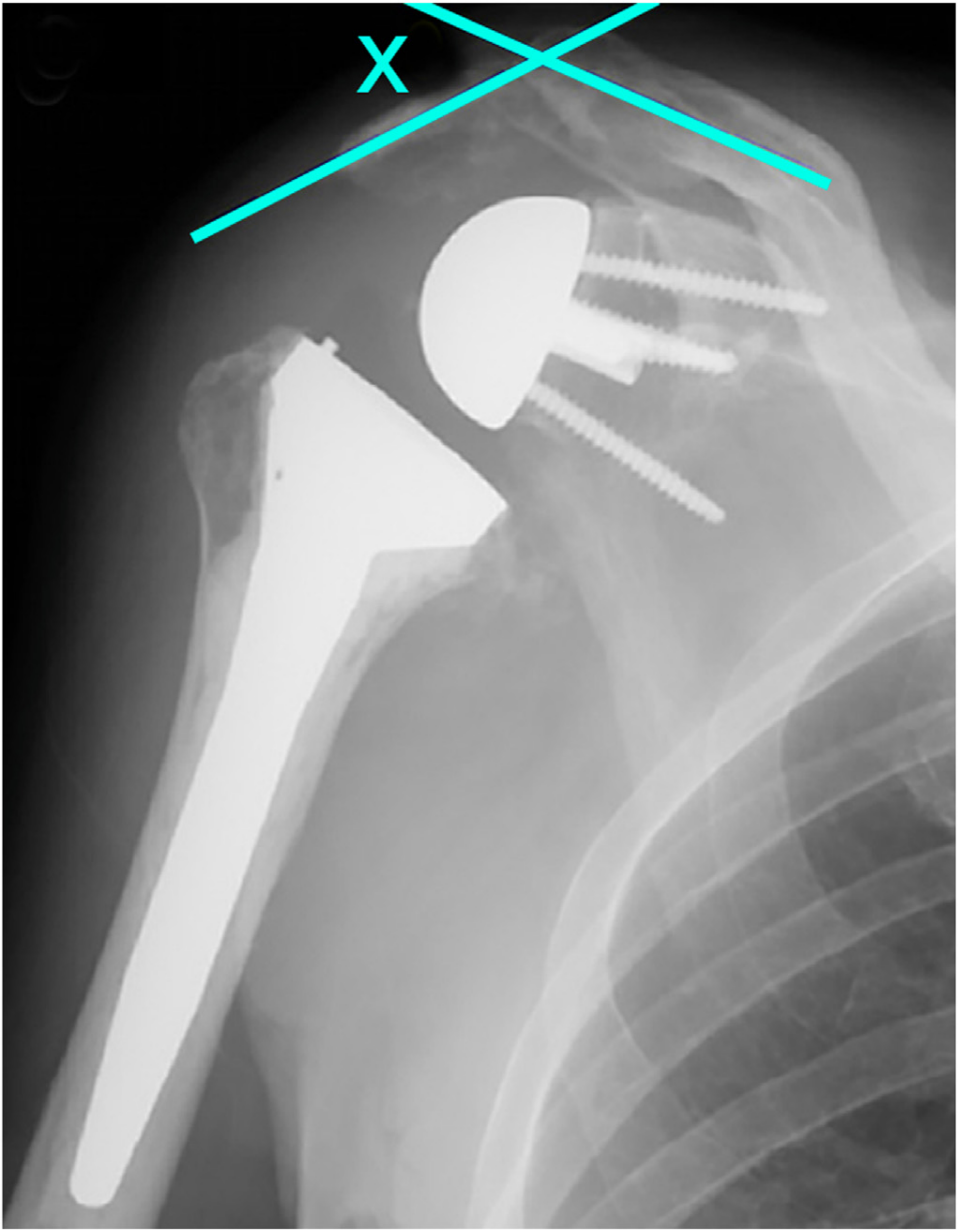
Inferior acromial tilting is measured on anteroposterior (AP) radiographs as the angle between the distal os segment and the proximal acromion. Aibinder et al has described tilting as any angle >5°.^2^

This review outlined inferior acromial tilting as the most common radiographic finding in this patient population (Fig. 3). While increased deltoid tension on the acromion in rTSA has been well described in the literature, those with underlying os acromiale are more susceptible given the lack of osseous continuity with an unfused segment. However, acromial tilting did not appear to affect the clinical outcomes of rTSA. Multiple studies have proposed that the lateral acromion is compensated for by the anterior and posterior portions of the deltoid inserting on an intact clavicle and scapular spine, respectively.^2^^,^^16^

While the rates of complications were generally low, it is important to be aware of the potential postoperative concerns of rTSA in patients with os acromiale. There was a wide range in the prevalence of postoperative pain related to the os acromiale (4%-27%). This may be due to several factors, including the different surgical techniques or implant designs selected for these patients. For example, humeral stem or glenosphere constructs that promote more distalization and deltoid tension may lead to more symptomatic and painful os fragments. Furthermore, the only surgical pearl that was suggested in this review was to avoid placement of a retractor on the acromion in patients with os acromiale.^5^ The studies included had great variability in terms of prosthesis selection at the time of surgery. The most common prosthesis designs included the Comprehensive (Zimmer-Biomet, Warsaw, IN, USA) and the Delta Xtend (DePuy, Raynham, MA, USA) systems but there was no clear correlation with specific outcomes that could be extrapolated from our findings.

This review was limited by the small number of studies and the heterogeneity of study designs. Most studies were retrospective in nature, with inherent limitations in terms of selection bias and confounding factors. Furthermore, the lack of standardized reporting, outcome measures, and follow-up time make it difficult to draw definitive conclusions. Future research should focus on prospective studies with larger sample sizes and standardized outcome measures. Evaluating different prosthesis designs, surgical techniques, and the effect on underlying os acromiale would provide more guidance to surgeons offering this type of surgery to patients. Lastly, studies investigating the impact of different os acromiale subtypes and the long-term outcomes of rTSA in these patients should be evaluated.

## Conclusion

This is the first systematic review to evaluate the effects of os acromiale in those undergoing rTSA. The presence of os acromiale does not appear to have a negative impact on the clinical outcomes after surgery and rTSA remains a safe and effective treatment option. Appropriate patient selection and education on the possible complications of underlying os acromiale shoulder should be considered in those undergoing rTSA.

## Disclaimers:

Funding: The authors confirm there were no outside funding or grants received for the completion of this study.

Conflicts of interest: The authors, their immediate families, and any research foundation with which they are affiliated have not received any financial payments or other benefits from any commercial entity related to the subject of this article.
